# Data on spatio-temporal representation of mineral N fertilization and manure N application as well as ammonia volatilization in French regions for the crop year 2005/06

**DOI:** 10.1016/j.dib.2018.09.119

**Published:** 2018-10-04

**Authors:** Sophie Génermont, Maharavo Marie Julie Ramanantenasoa, Karine Dufosse, Olivier Maury, Catherine Mignolet, Jean-Marc Gilliot

**Affiliations:** a UMR ECOSYS, INRA, AgroParisTech, Université Paris-Saclay, 78850, Thiverval-Grignon, France; b ADEME 20, avenue du Grésillé, BP 90406, 49004 Angers Cedex 01, France; c US Agroclim, INRA, Domaine Saint Paul, Site Agroparc, 84914 Avignon Cedex 9, France; d UR SAD, INRA, 88500 Mirecourt, France

## Abstract

The data presented in this article are related to the research article entitled “A new framework to estimate spatio-temporal ammonia emissions due to Nitrogen fertilization in France” (Ramanantenasoa et al., 2018) but are given with more details at a regional scale (NUTS2) in the objective to get them available for other research or applied studies. They concerns (i) the data implemented in the CADASTRE_NH_3_ framework and (ii) the data obtained using it, for crop year 2005/06. For the source data, the article focusses on the N fertilization practice management description, as this dataset is the most difficult to collect and to analyze in the objective of realistically representing the spatial and temporal variabilities needed in the framework.

**Specifications table**

**Table:** 

Subject area	*Agronomy, Atmospheric chemistry*
More specific subject area	*Crop N fertilization, manure application, ammonia volatilisation*
Type of data	*Tables*
How data was acquired	*Geo-referenced and temporally explicit databases, literature*
Data format	*Analyzed*
Experimental factors	*Brief description of any pretreatment of samples*
Experimental features	*Statistical analysis, Processing with a Geographical Information System, process-based model simulations*
Data source location	*France*
Data accessibility	*Part of the data* (Tables 1 and 2) *is available in the article*
	*The other data are provided as CSV format files in Supplementary material*
Related research article	*Ramanantenasoa M.M.J., Génermont S., Gilliot J.-M., Mignolet C., Bedos C., Mathias E., Eglin T., Makowski D., 2018. A process-based framework to estimate the spatio-temporal ammonia emissions due to nitrogen fertilization management. Sci. Total Environ., 645, 205-219.*https://doi.org/10.1016/j.scitotenv.2018.06.202.

**Value of the data**

• The data on fertilization can be used by other researchers or stake-holders or bodies in charge of national inventories to run other ammonia volatilization models, and to perform sensitivity analysis
• The data on fertilization can be used to run other models for spatial and temporal representation of other outcomes of N in the environment, for example nitrate lixiviation, nitrous oxide and nitric oxide emissions… using adapted models
• The spatio-temporal representation of the ammonia emission inventory could be compared with emissions inferred by atmospheric inversions using NH_3_ concentrations obtained from satellite observations. Data confrontation will help improving both methodologies
• The data should be used to improve the representation of N fertilization and ammonia emissions from agricultural activities in Chemical transport models. This will allow a better assessment of their impact on air quality and on N deposition to natural ecosystems, and improvement of air pollution peak forecasts.
• The data on fertilization and on volatilization can serve researchers, policy makers or local stake-holders for assessing the effectiveness of NH_3_ emission mitigation techniques, with regional specificities.

The authors welcome collaborative research in all these areas.

## 1 Data

In France, agriculture is responsible for 94% of ammonia (NH_3_) emissions with over 50% caused by nitrogen (N) fertilization. A new method was implemented to give a realistic representation of French ammonia emissions at different spatial and temporal scales: the “CADASTRE_NH_3_” framework. Its originality relies on the combined use of two types of resources: (i) a process-based ammonia volatilization model and (ii) geo-referenced and temporally explicit databases for soil properties, meteorological conditions and N fertilization management. The CADASTRE_NH_3_ framework is fully presented in a companion, full-length research article [10]. This article presents the source data used to describe the N fertilization practices encountered in crop year 2005/06 in France, and the outputs of this framework: the ammonia emissions and emission factors (Supplementry Table 4).

**Table 1:** Proportion of the three dominant mineral fertilizer types applied per region in crop year 2005/06.

Region	Proportion of ammonium nitrate	Proportion of nitrogen solution	Proportion of urea
	(%)	(%)	(%)
Alsace	81	5	14
Aquitaine	46	13	42
Auvergne	88	4	8
Basse-Normandie	66	22	11
Bourgogne	66	33	1
Bretagne	90	4	6
Centre	45	41	13
Champagne-Ardenne	22	74	4
Franche-Comté	93	7	0
Haute-Normandie	37	59	4
Ile-de-France	73	19	8
Languedoc-Roussillon	84	0	16
Limousin	97	1	2
Lorraine	35	58	7
Midi-Pyrénées	85	1	14
Nord-Pas-de-Calais	83	15	2
Pays-de-la-Loire	77	12	10
Picardie	45	51	3
Poitou-Charentes	52	25	22
Provence-Alpes-Côte-D’azur	97	0	3
Rhône-Alpes	86	1	13

**Table 2:** Organic manure properties.

Propertie	Unit	Cattle FYM^a^	Cattle slurry	Sheep FYM^a^	Pig slurry	Vinasse
Dry matter content	(g kg^−1^ fresh product)	200	90	300	65	620
Total N content	(g kg^−1^ fresh product)	5.5	4	9	5	25
TAN^b^ content	(g kg^−1^ fresh product)	0.55	1.6	0.9	3	5
pH	(dimensionless)	8	7	8	7.5	5
Fresh product density	(kg m^−3^)	750	1000	750	1000	1250

a FYM: Farm Yard Manure.

b TAN: Total Ammonical Nitrogen.

Supplementry Table 1 presents the mineral N fertilizer practices per crop and per region for the crop year 2005/06, i.e., the proportion of fields receiving mineral fertilization, and the classes of mineral N fertilization management: one to three classes are defined per crop per region for crop year 2005/06 and their respective contribution is quantified. Each fertilization management is characterized by the total number of fertilizations occurring on the same field, and each fertilization event is described by (i) the portion of the agricultural fields belonging to this class concerned by this fertilization, (ii) the amount of N applied, and (iii) the date range of the fertilization event.

Supplementry Table 2 presents the organic manure application practices per crop and per region for the crop year 2005/06, i.e., the proportion of fields receiving organic manure, and the organic manure application management per crop and per region. It is characterized by (i) the type of organic manure applied, (ii) the amount in N applied, and (iii) the date range when the application occurs. Five types of organic manure were selected: Cattle Farm Yard Manure (FYM), Sheep FYM, Cattle slurry, Pig slurry and Vinasse.

Supplementry Table 3 presents the crop area per region for the crop year 2005/06.

Supplementry Table 4 presents the data produced, i.e. the distribution of total nitrogen (N) and ammoniacal nitrogen (TAN) fertilizer use, annual ammonia (NH_3_) emissions and ammonia emission factors (EF) per fertilizer type and per region in France estimated using the CADASTRE_NH_3_ approach for 2005/06.

## 2 Experimental design, materials, and methods

The databases and the calculations processed are largely described in the companion article [10]. This article focuses on the data set the most challenging to build in a geo-referenced and temporally explicit way and on the output data.

N fertilization management data came from national survey of cultural practices for arable crops and grassland, conducted by the Department of Statistics and Forecasting of the French Ministry of Agriculture, for the crop year 2005/06, for 12 main crops (soft wheat, durum wheat, barley, grain maize, forage maize, oilseed rape, sunflower, peas, sugar beet, potato, grass leys and intensive permanent grasslands) in 21 French regions (Nomenclature of Territorial Units for Statistics: NUTS2) (data were unavailable for Corsica). No information was available for vegetables, fruits, vines, etc. which were not accounted for. The part of the fertilized fields was calculated considering the number of fields surveyed, and not the surface of the fields surveyed.

For mineral fertilizers, statistical calculations were carried out following Mignolet et al. [9]: a multivariate analysis including Multiple Correspondence Analysis (MCA) and Ascendant Hierarchical Classification (AHC) was performed on the three primary factorial axes based on Ward׳s distance. In fact, if the interval of application involved fewer than three plots, it was combined with the two weeks period adjacent to it, in order to comply with the restrictions related to the processing of these confidential data.

For organic manure, data were scarcer, and only one organic fertilizer application was described per crop and per Small Agricultural Regions (SAR: 713 French homogenous agricultural regions, with sizes ranging from 1096 to 440,650 ha).

In France in crop year 2005/06, the three most delivered mineral fertilizers were ammonium nitrate (NH_4_NO_3_), urea (CO(NH_2_)_2_), and N solution (50% N as NH_4_NO_3_, 50% N as CO(NH_2_)_2_) representing 81% of the total amount of N delivered as mineral fertilizer. Their relative proportions per region were derived from commercial fertilizer deliveries collected at the NUTS3 scale (French departments, ~ 600,000 ha) and provided annually by the *Union des Industries de la Fertilisation* (UNIFA). The information was smoothed over three crop years (2004/05, 2005/06 and 2006/07) to account for possible exceptional annual events impacting fertilizer sales.

Agronomic and physicochemical properties were calculated as the averages of the values found in French literature [3, 4, 6 and 7] for each type of organic manure represented.

Areas cultivated in crop year 2005/06 per crop per region were derived from the European Land Parcel Identification System (LPIS) for France [8], which was built within the framework of the Common Agricultural Policy (CAP) regulations.

Ammonia emissions were obtained for the crop year 2005/06 by running the process-based ammonia volatilization Volt’Air model [1], [2],5] on the geo-referenced and temporally explicit databases for N fertilization management described above, meteorological conditions, and soil properties. In regions where crops were not well represented, cultural practices were not surveyed: N fertilization management data were interpolated using the ones of the closer region where the crop was well represented. Spatial weather conditions, generated by the Système d’Analyse Fournissant des Renseignements Atmosphériques à la Neige (SAFRAN) model were delivered by Météo-France for years 2005 and 2006, on 8 km mesh grid. Soil spatial distribution was provided by the European Soil Data Center (ESDC) and soil properties by the Harmonized World Soil Database (HWSD) of the Food and Agriculture Organization (FAO). The runs were performed following a combinatory approach at the SAR level: the Volt’Air model was coupled with a scheduling tool written in python, to produce all input combinations over all SAR.

The total NH_3_ emission per crop per NUTS2 region was calculated as the sum of the NH_3_ emissions of organic manure and mineral N fertilizers of all SAR belonging to the same region. The national NH_3_ emission was calculated as the sum of the NH_3_ emissions of organic and mineral N fertilizer for all regions. Emissions can also be aggregated at smaller temporal scales ranging from hour to the whole crop year.

To match the EMEP/EEA Guide book formalisms and units, emission factors were calculated and expressed following different rules: for mineral fertilizers, ammonia volatilization EF were calculated as the ratio between (i) total N losses due to ammonia emissions per fertilizer type per region and (ii) total N applied for this fertilizer in this region; for organic manure, ammonia volatilization EF were calculated as the ratio in % of (i) total N losses due to ammonia emissions per manure type per region and (ii) Total Ammoniacal Nitrogen (TAN) applied for this manure in this region.

Same calculations were performed at the national scale and are shown in the related research paper [10].
