# Supplementary material for: Data on spatio-temporal representation of mineral N fertilization and manure N application as well as ammonia volatilization in French regions for the crop year 2005/06
Source: Data Brief. 2018 Oct 4;21:1119–24. doi: 10.1016/j.dib.2018.09.119 (PMC6230964; doi:10.1016/j.dib.2018.09.119)
Supplement: Supplementary file 1 — Supplementary material [file mmc1.docx]

Conflict of interest

All authors declare no conflict of interest.
